# Simple predictors of the re- occurrence of severe febrile neutropenia episode: a single-center retrospective cohort study in pediatric patients with malignant diseases

**DOI:** 10.3325/cmj.2019.60.20

**Published:** 2019-02

**Authors:** Silvije Šegulja, Alen Ružić, Dora Dujmić, Ksenija Baždarić, Jelena Roganović

**Affiliations:** 1 Department of Pediatrics, Hospital Thalassotherapia Crikvenica, Crikvenica, Croatia; 2 Department of Internal Medicine, Clinical Hospital Center Rijeka, Rijeka, Croatia; 3 Department of Internal medicine, University of Rijeka, Faculty of Medicine, Rijeka, Croatia; 4 Lawrence University, Appleton, WI, United States; 5 Department of Medical Informatics, University of Rijeka, Faculty of Medicine, Rijeka, Croatia; 6 Department of Pediatrics, Clinical Hospital Center Rijeka, Rijeka, Croatia; 7 Department of Pediatrics, University of Rijeka, Faculty of Medicine, Rijeka, Croatia

## Abstract

**Aim:**

To identify the risk factors of a repeated episode of severe febrile neutropenia (FN) and to build an accurate and easy-to-use predictive model.

**Methods:**

This single-center retrospective cohort study conducted at the Clinical Hospital Center Children’s Hospital Rijeka from January 1, 2008 to December 31, 2016 included pediatric patients with malignant diseases who experienced at least one FN episode. The association of the second severe FN episode appearance with relevant clinical and laboratory data was analyzed by logistic regression.

**Results:**

Out of 45 patients with one FN episode, 25 (56%) had severe FN and 11 (24%) had repeated severe FNs. Significant predictors of a repeated severe FN episode were the first FN episode duration of 9 or more days and red blood cells ≤3.0 × 10^12^/L. The predictive model constructed by crossing these two indicators had the accuracy of 87% (95% confidence interval [CI] 73%-94%), sensitivity of 82% (95% CI 53%-97%), and specificity of 88% (95% CI 79%-93%).

**Conclusion:**

The first FN episode duration and anemia are significantly associated with the risk for severe FN re-occurrence. These factors may be useful in the identification of children with cancer who are at high risk for adverse outcome at any future fever onset and may benefit from early intensive treatment.

The majority of pediatric malignances are treated by systemic combined chemotherapy, and approximately 80% of children are cured. Antineoplastic therapy adversely affects myelopoiesis and damages the integrity of gastrointestinal mucous membrane, leading to the invasion of colonizing bacteria and the development of a dangerous, quickly progressing systemic infection (1). Febrile neutropenia (FN) is the leading cause of immediate hospitalization in children with cancer and the most frequent complication of chemotherapy. It increases the morbidity and mortality due to serious infections (2-7).

In children with cancer, the beginning phase of a systemic bacterial infection, requiring early antibiotics treatment, has to be immediately differentiated from viral respiratory infections (8). However, the routine laboratory tests are not sensitive and specific enough for early detection of systemic inflammation. Some of these tests are also often time-consuming, such as blood cultures and antimicrobial susceptibility testing, which is still the gold standard for diagnostics and targeted therapy in systemic infections (9,10). Systems of prediction known as clinical decision rules (CDRs), on the other hand, do not include patient's individual characteristics into risk calculation and have not undergone external validation necessary for a wider clinical use (3,8). Monthly cycling antibiotic therapy emerged as a potential solution, but it needs to be further researched (11,12). The predictive value of serum concentrations of interleukin (IL)-6, IL-8, IL-10, and procalcitonin has also been analyzed, but the problem of early risk stratification has not been resolved (13-15). Data on predictive value of hemoglobin concentrations or red blood cell (RBC) count, known as indicators of myelosuppression, are similarly conflicting (16).

The overall outcome in children with malignant diseases can be improved by the reliable identification of individuals at high risk of systemic infections development at the time of fever onset (8,9,17). The International Consensus Statement for Core Outcomes and Definitions for Pediatric Fever and Neutropenia was agreed upon in 2015 to further develop this field but there are still no clear treatment algorithms (18). As the previously tested predictive models for any FN episode in children with cancer did not show clear clinical usefulness, we hypothesize that the focus should be on the prediction of repeated FN, and especially on repeated severe FN. Our aim was to determine which of the first FN episode clinical features could predict severe FN re-occurrence and facilitate clinical decision-making on early intensive antibiotic therapy in the next fever onset.

## Materials and methods

### Patients and study design

This retrospective single-center cohort study was conducted at the Clinical Hospital Center Rijeka, Location Kantrida, Department of Hematology and Oncology, Clinical Hospital Center Children’s Hospital Rijeka, Croatia. Data were collected from the hospital electronic records for patients treated from January 1, 2008 to December 31, 2016.

The inclusion criterion was the first episode of FN, while the exclusion criterion were incomplete data in medical history. We analyzed the medical records of 225 children with malignant diseases, while the final sample included 45 (20%) children who experienced at least one FN episode. Zero time was the time point of the beginning of the first FN episode.

The key outcome was a repeated episode of severe FN. The criteria for severe FN diagnosis were the absolute neutrophil count (ANC)≤500/mm^3^, the temperature ≥38.5°C (≥101.30°F), and duration longer than two days. Observed predictors were from three large groups of data: social, demographic and vitality, laboratory, and clinical data (18-21). The study protocol was approved by the Clinical Hospital Center Rijeka Ethics Committee and the University of Rijeka Faculty of Medicine Ethics Committee (No 19-03-0-013).

### Statistical analysis

Normality of data distribution was tested with the Kolmogorov-Smirnoff test. Data are presented as median and interquartile ranges. The predictive model was constructed in three steps. In the first step, the association of all variables with a severe FN repeated episode was assessed by univariate/unadjusted binary logistic regression. The variables that were significantly associated (*P* < 0.05) were included into the second step, when their association with severe FN repeated episode was analyzed by multivariate adjusted binary logistic regression. In the third step, two variables that showed significant partial/adjusted association with the criterion were combined into the final model. In predictive value analysis of the new model we calculated the overall accuracy, sensitivity, specificity, positive and negative likelihood ratios, and predictive values. The level of statistical significance was set to *P* < 0.05, and all confidence intervals were given on this level. The analyses were carried out using SPSS 17.0 (SPSS Inc., Chicago, IL, USA). Confidence intervals for proportions were determined using Statistics Calculator 3.0 (Stat Pac Inc., Bloomington, MN, USA). Measures of predictive value were calculated by John C. Pezzullo online calculator (*http://statpages.org/ctab2x2.html*).

## Results

Thirty-eight of 45 patients (84%) experienced a repeated FN, and the median number of FN episodes was 2 (range, 1-5). Twenty-five patients (55%) experienced severe FN and 11 (24%) experienced a second severe episode (Table 1).

**Table 1:** Patients' characteristics (N = 45)*

	N (%) or median (25-75)
Sociodemographic and vital parameters	
Sex	
male	25 (55.6)
female	20 (44.4)
Age at the diagnosis, years	5.0 (5.0-13.0)
BMI at first FN episode	16.0 (14.5-18.0)
Type of malignant disease	
acute lymphoblastic leukemia	17 (37.8)
neuroblastoma	5 (11.1)
non-Hodgkin’s lymphoma	5 (11.1)
other diagnosis^†^	18 (40.0)
Solid tumor	26 (57.8)
Metastatic disease	7 (15.6)
Bone marrow involvement	15 (33.3)
Broviac catheter	29 (64.4)
Duration of hospitalization in days	43 (23-77)
Febrile neutropenia (FN)	
at least one repeated episode	38 (84.4)
average number of episodes	2 (1-5)
severe FN	25 (55)
Repeated FN episode	
ANC	282 (17-500)
ANC≤500/mm^3^	27 (60.0)
temperature in °C	39.4 (38.8-39.8)
duration in days	2.5 (2.0-4.5)
duration ≥3 days	19 (42.2)
severe second FN episode	11 (24.4)

*FN – febrile neutropenia; ANC – absolute neutrophil count.

†Other diagnosis, two patients each: acute myeloid leukemia, aggressive fibromatosis, Ewing’s sarcoma, hepatoblastoma, Hodgkin lymphoma, medulloblastoma, Yolk sac tumor. Other diagnosis, one patient each: ganglioneuroblastoma, nephroblastoma, osteosarcoma, rhabdomyosarcoma.

In the first step of the univariate unadjusted binary logistic regression, six parameters were significantly associated with the severe repeated FN episode: lowest ANC≤300/mm^3^; duration of the first episode of 9 or more days; bone marrow suppression; RBC≤3.0 × 10^12^; hematocrit ≤0.25 (25%); and platelets ≤100 × 10^9^ (Table 2).

**Table 2:** Predictors associated (*P* < 0.05) with repeated episode of severe febrile neutropenia (FN) (N = 45)

	Severe FN repeated episode			OR_uni_ (95% CI)	OR_multi_ (95% CI)
N (%)	yes	no	total		
Lowest ANC at first FN episode					
ANC≤300/mm^3^	10 (38.5)	16 (61.5)	26 (100)	11.3 (1.21-261.6)	4.3 (0.26-71.59)
ANC>300/mm^3^	1 (5.3)	18 (94.7)	19 (100)		
Duration of the first FN episode					
≥9 days	6 (66.7)	3 (33.3)	9 (100)	12.4 (2.32-66.35)	30.4 (1.94-476.49)
≤8 days	5 (13.9)	31 (86.1)	36 (100)		
Bone marrow involvement					
yes	7 (46.7)	8 (53.3)	15 (100)	5.7 (1.32-24.54)	1.5 (0.16-13.69)
no	4 (13.3)	26 (86.7)	30 (100)		
Red blood cells					
≤3.0 × 10^12^	4 (80.0)	1 (20.0)	5 (100)	18.3 (1.76-189.63)	130.6 (1.3-13011)
>3.0 × 10^12^	7 (17.9)	32 (82.1)	39 (100)		
Hematocrit					
≤0.25	5 (71.4)	2 (28.6)	7 (100)	13.3 (2.08-85.41)	0.3 (0.00-9.77)
>0.25	6 (15.8)	32 (84.2)	38 (100)		
Platelets					
≤100 × 10^9^	5 (50.0)	5 (50.0)	10 (100)	4.8 (1.06-22.09)	1.0 (0.08-12.73)
>100 × 10^9^	6 (17.1)	29 (82.9)	35 (100)		

*OR_uni_ – univariate odds ratio for severe FN; OR_adj_ – multivariate, binary logistic regression adjusted odds ratio for severe FN; 95% CI – 95% confidence interval of odds ratio.

Two final significant predictors in the multiple regression were duration of the first episode of 9 or more days and RBC≤3.0 × 10^12^ (Table 2). These two variables combined accounted for approximately Nagelkerke R^2^ = 58% of the repeated severe FN episode variance. The final, simplified predictive model was constructed by crossing these two indicators (Table 3). High risk of severe FN repeated episode was indicated either by duration of the first episode of 9 or more days or by RBC≤3.0 × 10^12^, while low risk was indicated by the opposite values: duration of the first episode ≤8 days and RBC>3.0 × 10^12^. The new constructed predictor was significantly associated with the severity of repeated FN episode (Fisher exact test, *P* < 0.001) (Table 3). The overall accuracy of the model was 87% (95% CI 73%-94%), with the sensitivity of 82% (53%-97%) and the specificity of 88% (79%-93%). Positive likelihood ratio for repeated FN episode was 7.0 (95% CI 2.52-13.73). Negative likelihood ratio was 0.21 (95% CI 0.04-0.56).

**Table 3:** Predictive validity for a repeated episode of febrile neutropenia (FN) (n = 45)

	Repeated episode of FN			
	yes	no	total	
Estimated risk				
High*	9 (69.2)	4 (30.8)	13 (100)	positive predictive value 69% (45%-82%)^†^
Low	2 (6.3)	30 (93.8)	32 (100)	negative predictive value 94% (94%-99%)^†^
	sensitivity 82% (53%-97%)^†^	specificity 88% (79%-93%)^†^		

*High risk of FN repeated episode was indicated either by duration of the first episode ≥9 days or by RBC≤3.0 × 10^12^.

†Values in parentheses represent 95% confidence intervals.

## Discussion

This study showed two predictors of the re-occurrence of repeated severe FN: duration of the first FN episode of 9 or more days and RBC≤3.0 × 10^12^. Our model based on two predictors showed very good specificity, sensitivity, and negative predictive value and could be useful in clinical practice. To the best of our knowledge, these factors have not previously been shown to be significant predictors for severe FN. The duration of the first episode of 9 or more days could indicate slower hematopoietic recovery, while the lower RBC count can be a consequence of myelosuppression or bone marrow infiltration. As the pathophysiology of FN is multifactorial, other possible causes have also to be taken into consideration (15).

Considerable evidence has been published on different single and combined predictors for severe FN. C reactive protein ≥90 mg/L, hypotension, platelet number ≤50 000 /mm^3^, relapsed leukemia, and chemotherapy in the last 7 days were shown to be the predictors for severe bacterial infection (19), while intensive chemotherapy, shorter time-to-diagnosis, presence of CVC and previous FN were shown to be predictors for development of FN and FN with bacteremia (20).

Our study found several significant univariate predictors. ANC≤300/mm^3^ was a good predictor of severe FN episodes, which is consistent with the results by Ammann et al (15). Also, hematocrit ≤0.25 was a good predictor, pointing toward bone marrow suppression or bone marrow infiltration as a possible explanation for FN susceptibility. Bone marrow involvement in malignant disease was also univariately significant, while in other studies it demonstrated multivariate significance (15,20). Platelet count ≤100 × 10^9^/L was a good univariate predictor in our study, as well as in other studies (20), even in multivariate models (19,22). Other parameters investigated in this study were found to be non-significant by other authors (19,22).

Disease-specific factors depending on the type of malignant disease, type and dose of chemotherapy, comorbidities, early complications, and other patients' characteristics make the risk assessment by general score systems problematic (23-25). Only one of the available CDRs is methodologically suitable, however, it was developed and validated in South America, making its application in other parts of the world questionable (26). Also, while the greatest part of CDRs identify children at low risk of severe infection, only one of them aims at diagnosing severe bacterial infection (26-29). Better risk-stratification of patients based on our model could lead to a less intensive treatment in low-risk patients, which carries a lower risk of side effects and hospital acquired infections, lower toxicity risk and the risk of antibiotic resistance development, better quality of life for the child and the family, as well as lower costs. However, identifying children at low risk cannot have the advantage over the identifying children at high risk who can be successfully cured by an early intensive antimicrobial therapy.

Our proposal is oriented toward the general risk assessment and includes both of these groups. Also, our results seem to be more appropriate for severe FN prediction in pediatric oncologic patients than the Systemic Inflammatory Response Syndrome (30), which is used for sepsis diagnosis but is generally questionable in oncologic patients, since it was based on studies that did not include a considerable number of patients with tumors and neutropenia. MASCC risk score by the Association for Supportive Care in Cancer, which assesses the risk of adverse outcome in oncologic patients, is also not suitable for children with tumors, since it shows gaps in evidence and requires numerous tests to be made (CRP, procalcitonin, venous blood lactate, proteinemia and phosphatemia evaluation, blood lactate, antithrombin, and VIIa factor levels, and chest x-ray) (31).

The results of this study need to be validated on larger patient populations. Also, the number of our patients did not allow sub-analyses according to malignant disease type, phase, chemotherapy type, and chemotherapy intensity. Long time period in which the study was conducted could have influenced the final results, and data on the time-to-antibiotic administration were not available.

In conclusion, we propose a predictive model for the second FN episode based on a new combination of two easily accessible risk factors. This model could be used for risk assessment in different malignant diseases, chemotherapy types, and chemotherapy intensities. Our approach might represent a step toward an individually tailored FN therapy and the prediction of repeated severe FN episode in pediatric oncology, facilitating decision-making on inpatient vs outpatient management.

## References

[1] Freifeld AG, Bow EJ, Sepkowitz KA, Boeckh MJ, Ito JI, Mullen CA (2011). Clinical practice guideline for the use of antimicrobial agents in neutropenic patients with cancer: 2010 Update by the Infectious Diseases Society of America.. Clin Infect Dis.

[2] Culakova E, Thota R, Poniewierski MS, Kuderer NM, Wogu AF, Dale DC (2014). Patterns of chemotherapy-associated toxicity and supportive care in US oncology practice: a nationwide prospective cohort study.. Cancer Med.

[3] Muller EL, Walkovich KJ, Mody R, Gebremariam A, Dais MM (2015). Hospital discharges for fever and neutropenia in pediatric cancer patients: United States, 2009.. BMC Cancer.

[4] Kuderer NM, Dale DC, Crawford J, Cosler LE, Lyman GH (2006). Mortality, morbidity, and cost associated with febrile neutropenia in adult cancer patients.. Cancer.

[5] Osmani AH, Jabbar AA (2017). Gangwani, Hassan B. Outcomes of high risk patients with febrile neutropenia at a tertiary care center.. Asian Pac J Cancer Prev.

[6] Kumar A, Roberts D, Wood KE, Light B, Parrillo JE, Sharma S (2006). Duration of hypotension before initiation of effective antimicrobial therapy is the critical determinant of survival in human septic shock.. Crit Care Med.

[7] Salstrom JL, Coughlin RL, Pool K, Bojan M, Mediavilla C, Schwent W (2015). Pediatric patients who receive antibiotics for fever and neutropenia in less than 60 min have decreased intensive care needs.. Pediatr Blood Cancer.

[8] Haeusler GM, Sung L, Ammann RA, Phillips B (2015). Management of fever and neutropenia in paediatric cancer patients: room for improvement?. Curr Opin Infect Dis.

[9] Haeusler GM, Levene I (2015). What are the risk factors for antibiotic resistant Gram-negative bacteraemia in children with cancer?. Arch Dis Child.

[10] Gies F, Tschiedel E, Felderhoff-Müser U, Rath PM, Steinmann J, Dohna-Schwake C (2016). Prospective evaluation of SeptiFast Multiplex PCR in children with systemic inflammatory response syndrom under antibiotic treatment.. BMC Infect Dis.

[11] Korber F, Zeller I, Grunstaudl M, Willinger B, Apfalter P, Hirschl AM (2017). SeptiFast versus blood culture in clinical routine – a report on 3 years experience.. Wien Klin Wochenschr.

[12] Teranishi H, Koga Y, Nishio H, Kato W, Ono H, Kanno S (2017). Clinical efficacy of cycling empirical antibiotic therapy for febrile neutropenia in pediatric cancer patients.. J Infect Chemother.

[13] Sahbudak BZ, Karadas ON, Sen S, Yilmaz KD, Azarsiz E, Avdemir S (2017). Diagnostic accuracy of interleukin-6, interleukin-8, and interleukin-10 for predicting bacteremia in children with febrile neutropenia.. Turk J Haematol.

[14] Hemming V, Jakes AD, Shenton G, Phillips B (2017). Prospective cohort study of procalcitonin levels in children with cancer presenting with febrile neutropenia.. BMC Pediatr.

[15] Ammann RA, Bodmer N, Hirt A, Niggli FK, Nadal D, Simon A (2010). Predicting adverse events in children with fever and chemotherapy-induced neutropenia: the prospective multicenter SPOG 2003 FN study.. J Clin Oncol.

[16] Ammann RA, Niggli FK, Leibundgutt K, Teuffel O, Badmer N (2014). Exploring the association of hemoglobin level and adverse events in children with cancer presenting with fever in neutropenia.. PLoS One.

[17] Lucas AJ, Olin JL, Coleman MD (2018). Management and preventive measures for febrile neutropenia.. P&T.

[18] Haeusler GM, Phillips RS, Lehrnbecher T, Thursky KA, Sung L, Ammann RA (2015). Core outcomes and definitions for pediatric fever and neutropenia research: a consensus statement from an international panel. International consensus recommendations for core outcomes and definitions for pediatric fever and neutropenia research.. Pediatr Blood Cancer.

[19] Santolaya ME, Alvarez AM, Becker A, Cofre J, Enriquez N, O’Ryan M (2001). Prospective, multicenter evaluation of risk factors associated with invasive bacterial infection in children with cancer, neutropenia and fever.. J Clin Oncol.

[20] Wicki S, Keisker A, Aebi C, Leibundgut K, Hirt A, Ammann RA (2008). Risk prediction of fever in neutropenia in children with cancer: a step towards individually tailored supportive therapy?. Pediatr Blood Cancer.

[21] Rackoff WR, Gonin R, Robinson C, Kreissman SG, Breitfeld PB (1996). Predicting the risk of bacteremia in children with fever and neutropenia.. J Clin Oncol.

[22] Santolaya ME, Alvarez AM, Aviliés CL, Becker A, Cofre J, Enriwuez N (2002). Prospective evaluation of a model of prediction of invasive bacterial infection risk among children with cancer, fever, and neutropenia.. Clin Infect Dis.

[23] Klaassen RJ, Goodman TR, Pham B, Doyle JJ (2000). “Low-risk” prediction rule for pediatric oncology patients presenting with fever and neutropenia.. J Clin Oncol.

[24] Ahmed N, El-Mahallawy HA, Ahmed IA, Nassif S, El-Beshlawy A, El-Haddad A (2007). Early hospital discharge versus continued hospitalization in febrile pediatric cancer patients with prolonged neutropenia: A randomized, prospective study.. Pediatr Blood Cancer.

[25] Oberoi S, Das A, Trehan A, Ray P, Bansai D (2017). Can complications in febrile neutropenia be predicted? Report from a developing country.. Support Care Cancer.

[26] Delebarre M, Macher E, Mazingue F, Martinot A, Dubos F (2014). Which decision rules meet methodological standard sin children with febrile neutropenia? Results of a systematic review and analysis.. Pediatr Blood Cancer.

[27] Phillips RS, Lehrnbecher T, Alexander S, Sung L (2012). Updated systematic review and meta-analysis of the performance of risk prediction rules in children and young people with febrile neutropenia.. PLoS One.

[28] Vedi A, Pennington V, O’Meara M, Stark K, Senner A, Hunstead P (2015). Management of fever and neutropenia in children with cancer.. Support Care Cancer.

[29] Moreira DG, Costello JT, Brito CJ, Adamczyk JG, Ammer K, Bach AJE (2017). Thermographic imaging in sports and exercise medicine: A Delphi study and consensus statement on the measurement of human skin temperature.. J Therm Biol.

[30] Tam CS, O’Reilly M, Andresen D, Lingaratnam S, Kelly A, Burbury K (2011). Use of empiric antimicrobial therapy in neutropenic fever. Australian Consensus Guidelines 2011 Steering Committee.. Intern Med J.

[31] Thursky KA, Worth LJ (2015). Can mortality of cancer patients with fever and neutropenia be improved?. Curr Opin Infect Dis.

